# Inequalities in the receipt of healthcare practitioner counseling for adults after COVID-19 in southern Brazil

**DOI:** 10.1186/s12889-023-15914-2

**Published:** 2023-06-07

**Authors:** Juliana Quadros Santos Rocha, Rinelly Pazinato Dutra, Yohana Pereira Vieira, Suele Manjourany Silva Duro, Mirelle de Oliveira Saes

**Affiliations:** 1 Postgraduate Programme in Health sciences, Federal University of Rio Grande, Visconde de Paranaguá, 102, bairro Centro, Rio Grande, Rio Grande do Sul 96203-900 Brazil; 2 Postgraduate Programme in Public Health, Federal University of Rio Grande, Rio Grande, Rio Grande do Sul Brazil; 3 Postgraduate Programme in Nursing, Federal University of Pelotas, Pelotas, Brazil

**Keywords:** Counseling, Disparities in health levels, COVID-19

## Abstract

Health counseling is a prevention and health promotion action, especially in the context of a pandemic, for both preventing disease and maintaining health. Inequalities may affect receipt of health counseling. The aim was to provide an overview of the prevalence of receiving counseling and to analyze income inequality in the receipt of health counseling. Methods: This was a cross-sectional telephone survey study with individuals aged 18 years or older with diagnosis of symptomatic COVID-19 using RT-PCR testing between December 2020 and March 2021. They were asked about receipt of health counseling. Inequalities were assessed using the Slope Index of Inequality (SII) and Concentration Index (CIX) measures. We used the Chi-square test to assess the distribution of outcomes according to income. Adjusted analyses were performed using Poisson regression with robust variance adjustment. Results: A total of 2919 individuals were interviewed. Low prevalence of health counseling by healthcare practitioner was found. Participants with higher incomes were 30% more likely to receive more counseling. Conclusions: These results serve as a basis for aggregating public health promotion policies, in addition to reinforcing health counseling as a multidisciplinary team mission to promote greater health equity.

**Supplementary Information:**

The online version contains supplementary material available at 10.1186/s12889-023-15914-2.

## Introduction

The COVID-19 pandemic has caused notorious social, economic and health impacts [1–3]. According to the literature, the significant number of deaths caused by sars-cov-2 infection is due to its high transmission capacity, and the socially disadvantaged are highly vulnerable to infection, demonstrating that the pandemic exacerbates social inequalities [4].

Knowledge gained from previous pandemics has shown that care measures such as hand hygiene, mask use, social distancing and vaccination are effective measures for slowing the spread of viruses [5]. In addition, counseling on a healthy lifestyle (healthy eating, practicing physical activity, and adequate sleep) is an essential strategy for behavior change, being important for preventing disease, maintaining health and avoiding complications caused by sars-cov-2 infection [6, 7].

Counseling is a powerful and recognized effective health promotion practice for adherence to healthy behaviors and habits, and consequently, for improving people’s quality of life, and it is a comprehensive process of listening, understanding, support, and individual or collective help [8, 9]. Studies point out that individuals who receive counseling present benefits associated with the improvement of behavioral aspects, greater engagement in healthy habits and a better perception of overall health [9, 10]. Applied in several areas of knowledge, counseling has proven to be a promising strategy for encouraging people to plan and make decisions about their health-disease-care process and modify behaviors, but it is still not very prevalent in health services [11–13]. No studies are known about people with covid-19 receiving counseling during the pandemic, but literature outside this context shows prevalence of counseling regarding healthy eating at around 40.0–59.0% in primary care [14]. As for practicing physical activity, a review study shows counseling prevalence ranging between 20.0% and 59.4%, being more frequent among women, the chronically ill and those with more education [7, 12, 14]. In turn, inequality is historical in Brazil and has been made worse by the context of the pandemic [15] and, although most studies reflect health inequalities through use of and access to health services [16], little is known about counseling indicators.

In this sense, given that counseling is an indispensable action for prevention and health promotion, this study aims to provide an overview of the prevalence of receiving counseling and analyze income inequality in receiving counseling for measures to protect and combat the new coronavirus (hand hygiene, use of masks, social distancing and vaccination), counseling on a healthy lifestyle (healthy eating, physical activity and healthy sleep) in individuals with covid-19 in a city in southern Brazil.

## Methodology

This is a cross-sectional study carried out in the city of Rio Grande in the far south of the state of Rio Grande do Sul/RS. Rio Grande is a port city, covering 2,817 km², with a population of 212,881 inhabitants [17]. This study protocol was approved by the Health Research Ethics Committee (CEPAS) of the Federal University of Rio Grande (FURG) (Certificate of Submission for Ethical Appraisal No. 39081120.0.0000.5324).

Our target population consisted of all individuals aged 18 years or older, infected between December/2020 and March/2021, diagnosed with covid-19 by RT-PCR, living in the urban area of the city of Rio Grande (Rio Grande do Sul, Brazil), who presented symptoms during infection and were followed by the epidemiological surveillance of the municipality. We excluded individuals living in rural areas, without cognitive capacities to answer the instrument and with no caregiver/guardian to answer for them, without telephone contact and address available in the epidemiological surveillance register, deprived of freedom, and who were no longer residing in the city during data collection. Individuals who were not located after five attempts (one via phone contact, one via WhatsApp and three home visits) were considered losses and individuals who denied participating in the interview were considered refusals.

Contact was made with the epidemiological health surveillance service of the municipality of Rio Grande to identify adults and elderly people infected with COVID-19 in the investigated period, thus creating a list of individuals with positive RT-PCR and their respective data (name, address, telephone number and presence of symptoms). Data collection by telephone was done based on the list of individuals who were eligible for the study. Data collection was carried out by previously trained interviewers, who underwent a selection process and later underwent training and qualification that lasted for a total of 24 h. In addition to telephone interviews, home visits were offered, for face-to-face data collection, if necessary.

After reading the Free and Informed Consent form, individuals who voluntarily agreed to participate in the research were interviewed. Data collection took place from July to October 2021. Questionnaires were collected electronically (tablet) using the REDcap program and using smartphones for telephone calls. Calls were recorded to ensure the safety of the researcher and interviewee, using a free cell phone application (Callmaster), stored in an e-mail account. The questionnaire took approximately 20 min to answer.

The questionnaire contained questions on demographic information (sex, age, ethnicity), socioeconomic status (education, marital status and economic class), previous diseases (hypertension, diabetes mellitus, respiratory diseases, heart disease) and use of health services (primary, secondary, urgency and emergency and tertiary).

Participants were asked about receiving health counseling for: (1) hand hygiene, mask use, social distancing; (2) practicing physical activity; (3) healthy eating; (4) sleep habits; and (5) vaccination against COVID-19. The questions relating to these variables were asked as follows, with the reply options being “yes” and “no”. “Since you were infected with COVID-19 until now, have you received any information or recommendations from healthcare practitioner (doctors, nurses, community workers, dentists and others) about:

- Preventive measures and measures to combat the novel coronavirus (Sars-CoV-2), such as: hand hygiene, use of a mask, social distancing?
- Practicing physical activity?
- The importance of maintaining a healthy diet (reducing intake of salt, sweets and fats)?
- Maintaining healthy sleeping habits such as sleeping at least 7 h a night?
- The importance of vaccination against COVID-19?”

Three synthetic outcomes were built based on the above individual outcomes, as follows:

- Healthy lifestyle counseling (food, sleep and physical activity);
- Counseling on COVID-19 prevention (hand hygiene, use of a mask, social distancing and vaccination);
- Counseling for all five of the above guidelines.

As such, eight counseling outcomes were considered in the analyses: (1) preventive measures and measures to combat the novel coronavirus; (2) practicing of physical activity; (3) importance of maintaining a healthy diet; (4) maintaining healthy sleeping habits; (5) importance of vaccination against covid-19; (6) healthy lifestyle; (7) covid-19 prevention; and (8) receiving all five items of counseling. Each outcome was dichotomized into “yes” and “no” and, for outcome 8, “yes” was considered to be a positive response to all outcomes.

The independent variable is income in BRL − 0-1000/1001–2000/2001–4000/ 4001 or more and the adjustment variables are: demographic (sex - male and female, age − 18 to 59 years and 60 years or older, and skin color - white and black/brown), socioeconomic (marital status - married/lives with a partner and single/ separated/widowed, schooling - elementary education / high school education / higher education) and nutritional (body mass index - underweight/normal weight and overweight/obese). The SII represents the absolute difference, in predicted values, of an indicator of health among the most favored and least favored individuals in terms of socioeconomic indicators, taking into account the entire distribution of the stratifier through a regression model. It’s calculated as the absolute difference in health indicator value between the highest (score of 1) and the lowest (score of 0) value of the socioeconomic indicator classification. The SII ranges from − 100 to 100 p.p. (percentage points), with values close to zero expected in the absence of inequality [18]. The CIX is analogous to the Gini index - it varies from − 1 to + 1, assumes zero to be equality, and the further the values ​​are from zero, the greater the relative inequality. Thus, positive values ​​indicate pro-rich differences and negative values ​​indicate pro-poor differences. Some studies, like ours, also present the CIX as values ​​multiplied by 100, for reasons of data visualization, together with measures of absolute inequalities, without changing their interpretation [18].

Descriptive data are presented as proportions and 95% confidence intervals (95%CI). We used the Chi-square test to assess the distribution of outcomes according to income. Adjusted analyses were performed using Poisson regression with robust variance adjustment. All associations with 95%CI without overlap between categories were considered statistically significant. Data were analyzed using the Stata 17.1 statistical package.

## Results

A total of 4014 participants were diagnosed with covid-19 in the period predetermined by the study. After applying the selection criteria, 3822 of them were eligible for the study (192 were excluded due to incomplete data and/or not showing symptoms during the acute phase of infection). After losses and refusals (631 and 272, respectively), 2919 individuals were interviewed (76.4% of those eligible). Of these, 24.7% used at least one health service. The sample consisted mostly of women (58.6%), individuals between 18 and 59 years old (83.3%), of white skin color (77.9%) and who were married or living with a partner (60.6%). As for education and income, 44.2% had completed high school and 38.9% had monthly income of between BRL 1000–2000. In addition, 73.3% were overweight/obese (Table 1).

**Table 1:** Description of the sample according to sex, age, skin color, marital status, education, income and BMI of individuals with COVID-19 (December 2020 to March 2021) in the municipality of Rio Grande, Rio Grande do Sul, 2022 (n = 2919)

Variable	%	n
**Sex**		
Female	58.6	1711
Male	41.4	1208
**Age group**		
18 to 59 years	83.3	2420
60 years or more	16.7	484
**Skin color**		
White	77.9	2254
Black/brown	22.1	640
**Marital status**		
Married/lives with partner	60.6	1757
Single/separated/widowed	39.4	1144
**Education**		
Never studied	0.52	15
Elementary education	24.9	713
High school education	44.2	1264
Higher education	30.4	871
**Income (BRL)**		
0-1000	26.1	668
1001–2000	38.9	995
2001–4000	23.6	604
4000 or +	11.3	288
**Body mass index**		
Low/normal weight	26.7	757
Overweight/obesity	73.3	2076

**Fig. 1:**
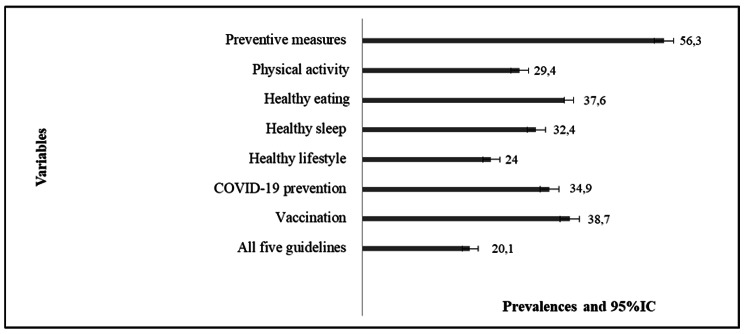
Prevalence of counseling among individuals with COVID-19 (December 2020 to March 2021) in the municipality of Rio Grande, Rio Grande do Sul, 2022 (n = 2919)

Regarding the occurrence of counseling, 56.3% of respondents reported having received counseling for preventive measures and measures to combat covid-19, 29.4% for practicing physical activity, 37.6% for healthy eating, 32.5% for healthy sleep and 38.7% for covid-19 vaccination. Regarding the prevalence of the synthetic outcomes, we found that about a quarter of the sample received counseling for a healthy lifestyle (24.0%), 34.0% for prevention of covid-19 and only 20.1% of the interviewed individuals reported receiving counseling for all five guidelines investigated (Fig. 1).

Table 2 shows the prevalence of outcomes in relation to income. The lowest prevalence of counseling was for all guidelines among those with income between BRL 0–1,000.00 (15.7%; 95% CI13.1-18.6), while prevalence was highest for protective measures for individuals with income between BRL 2,001.00–4,000.00 (58.1%; 95%CI 54.1–62.0). Virtually all surveyed outcomes showed a progressive increase in the prevalence of counseling as income increased, with differences between the extremes ranging between 9 and 12.7% points (counseling for all guidelines and for covid-19 vaccination, respectively).

In the analysis adjusted by income groups, the probability of receiving counseling was about 30.0% higher in the wealthiest groups for healthy lifestyle (PR:1.30, 95%CI 1.05; 1.63), healthy eating (PR:1.30; 95%CI 1.07; 1.59), physical activity (PR 1.29 95%CI 1.07; 1.56) and healthy sleeping (PR:1.30 95%CI 1.04;1.61). With regard to receiving counseling on covid-19 prevention and vaccination, prevalence was around 40% pro-rich (PR: 1.47 95%CI 1.20; 1.80, PR: 1.37; 95%CI 1.14; 1.65, consecutively). Finally, prevalence of counseling for all guidelines was also around 50% higher in individuals with higher incomes (PR: 1.53; 95%CI 1.12; 2.09) (Table 3).

Regarding the magnitude of income inequalities, counseling on healthy lifestyle and covid-19 prevention was more concentrated among the richest, with absolute differences between the prevalence of the first and fourth income groups ranging from 11.0 to 12.9 p.p. represented by the SII (SII:11.0, 95%CI 5.6;16.5; SII:12.9; 95%CI 6.8;19.0, respectively). In relation to each item of counseling, all of them showed greater convergence for the most well-off. In the same sense, relative inequalities (CIX) were significantly higher for counseling on all items (CIX: 8.8; 95%CI 4.7; 13.0) (Table 4).

**Table 2:** Distribution of counseling according to the income of individuals with COVID-19 (December 2020 to March 2021) in the municipality of Rio Grande, Rio Grande do Sul, 2022 (n = 2919).

Variables	0-1000		1001–2000		2001–4000		4000 or +	
	%	95%CI	%	95%CI	%	95%CI	%	95%CI
Healthy eating	33.1	(29.6; 36.8)	36.6	(33.6; 39.6)	41.8	(37.9; 45.8)	42.6	(36.9; 48.5)
Physical activity	24.3	(21.1; 27.7)	27.9	(25.2; 30.8)	34.2	(30.5; 38.1)	35.9	(30.5; 41.7)
Healthy sleeping	28.5	(25.2; 32.1)	30.7	(27.9; 33.7)	35.5	(31.8; 39.4)	39.2	(33.7; 45.1)
Healthy lifestyle	19.6	(16.4; 22.8)	22.3	(19.8; 25.0)	28.0	(24.5; 31.8)	29.7	(24.6; 35.3)
Coronavirus protective measures	55.6	(51.8; 59.4)	55.5	(52.3; 58.4)	58.1	(54.1; 62.0)	57.4	(51.5; 63.0)
Vaccination	33.4	(29.9; 37.1)	36.7	(33.8; 39.8)	42.0	(38.1; 46.0)	46.1	(40.4; 52.0)
COVID-19 prevention	29.8	(26.5; 33.4)	33.5	(30.6; 36.5)	37.9	(34.0; 41.8)	41.9	(36.3; 47.7)
All five guidelines	15.7	(13.1;18.6)	18.4	(16.0;20.9)	23.9	(20.6;27.5)	24.7	(20.0;30.1)

**Table 3:** Adjusted analysis of counseling and income of individuals with COVID-19 (December 2020 to March 2021) in the municipality of Rio Grande, Rio Grande do Sul, 2022 (n = 2919).

Variables	Income			
	0-1000	1001–2000	2001–4000	4000 or +
Healthy eating	1	1.06 (0.92; 1.23)	1.25 (1.07; 1.47)	1.30 (1.07; 1.59)
Physical activity	1	1.11 (0.94; 1.33)	1.29 (1.07; 1.56)	1.32 (1.04; 1.66)
Healthy sleeping	1	1.04 (0.89; 1.23)	1.18 (0.99; 1.41)	1.30 (1.04;1.61)
Healthy lifestyle	1	1.10 (0.90;1.34)	1.30 (1.05; 1.63)	1.35 (1.03; 1.77)
Coronavirus protective measures	1	1.02 (0.93;1.12)	1.08 (0.97; 1.20)	1.07 (0.93; 1.23)
Vaccination	1	1.09 (0.95; 1.26)	1.25 (1.07; 1.46)	1.37 (1.14; 1.65)
COVID-19 prevention	1	1.13 (0.97; 1.31)	1.30 (1.10; 1.54)	1.47 (1.20; 1.80)
All Guidelines	1	1.16 (0.92; 1.46)	1.47 (1.14; 1.90)	1.53 (1.12; 2.09)

Adjusted for sex, age, skin color, marital status, education and body mass index.

**Table 4:** Relative inequality index (CIX) and absolute inequality (SII) of counseling according to the income of individuals with COVID-19 (December 2020 to March 2021) in the municipality of Rio Grande, Rio Grande do Sul, 2022 (n = 2919).

Variables	Concentration index	95%CI	Slope Index of Inequality	95%CI
Healthy eating	4.4	(1.8; 7.1)	9.4	(3.2; 15.7)
Physical activity	5.3	(2.1; 8.6)	11.3	(5.0; 17.2)
Healthy sleeping	5.2	(2.2; 8.2)	10.7	(4.6; 16.7)
Healthy lifestyle	6.7	(3.0; 10.5)	11.0	(5.6; 16.5)
Coronavirus protective measures	0.2	(-1.5; 2.1)	2.3	(-4.2; 8.7)
Vaccination	5.0	(2.4; 7.2)	13.8	(7.6; 20.1)
COVID-19 prevention	5.5	(2.6; 8.4)	12.9	(6.8; 19.0)
All five guidelines	8.8	(4.7; 13.0)	11.6	(6.4; 16.8)

## Discussion

The data presented in this study show low prevalence of health counseling by healthcare practitioners, with counseling on coronavirus protective measures having the highest prevalence and counseling on physical activity having the lowest (56.3% and 29.4%, respectively). We found that the probability of participants receiving counseling was 30% higher among those with higher incomes, and this difference occurred progressively. As for the magnitude of inequalities in relation to income for each item of counseling, all of them showed a higher concentration among the wealthier.

Recommendations related to coronavirus protective measures and measures to combat covid-19 were low, despite their being recognized as effective methods [19]. Health managers and governments have programmed a series of interventions to stop the rapid evolution of the pandemic in order to avoid the overburdening of health systems, allow timely treatment of serious complications and prevent deaths [20]. It is important to note that during the data collection period, when the proportion of vaccine was insufficient for the entire Brazilian population, these behaviors were the only effective strategies to reduce contagion [20]. Counseling could be a way of reducing fake news that has brought so much harm to the health of the population, promoting ignorance and jeopardizing the credibility of the Brazilian National Health System [21]. Finally, counseling could be a countermeasure to the scientific denialism proposed by the current Federal Government, disregarding the number of deaths and the negative repercussions caused by the pandemic [22].

Social isolation and the consequences associated with the covid-19 pandemic, which include reduced physical activity, increased unhealthy eating habits, and increased levels of stress that cause sleep disturbances, expose people to deleterious physical and mental effects [23]. Due to the importance of these recommendations at an unfavorable moment for the health of the population, such as the covid-19 pandemic, counseling should be strongly disseminated by healthcare practitioner to individuals on a larger scale. However, it can be seen that data on the occurrence of some counseling before the covid-19 pandemic are similar to or lower than our results. In Brazil as a whole, prevalence of counseling on healthy eating was around 40.0–59.0% in primary care [14], 29.0% and 38.0% in a national population-based study [13] and 19.9% ​​in a population-based study conducted in Southern Brazil [12] while for physical activity it was 20.0–59.4% and 28.9% among adults and 38.9% among the elderly in a population survey [24]. Prevalence of healthy sleep counseling is not described in the literature [25].

The importance of counseling lies in the benefit of the actions themselves. Adequate nutrition through macronutrients and micronutrients helps correct organ functioning and disease prevention, in addition to having an impact on the prognosis of those infected [26]. Regarding practicing physical activity, promotion of physical activities at home with the help of the internet and other media is necessary, since regular activities have positive effects on functional capacity, reduction of blood pressure levels and depression, improvement of cognitive, muscular, cardiovascular and immunological functions [27, 28]. Finally, mental health issues have become a global concern during the pandemic and sleep disorders are the main mental health issues associated with increased psychosocial stressors [29]. Thus, recommendations for adequate sleeping habits are necessary, given the strong relationship with emotional issues, physical health and well-being acting on the immune, metabolic, endocrine systems and inflammatory processes, essential for mental well-being and quality of life [30, 31].

In relation to social inequalities, greater vulnerability can be observed in relation to the poorest. Despite the progress Brazil has made with health indicators as a result of the country’s development and the project to universalize access to health [32], it still persists among the 10 countries with the greatest inequality in the world [33]. Progress with health is also uneven, with less progress among subgroups in the worst socioeconomic position [34]. Economic inequality can play an important role in the impact of COVID-19 in relation to the increase in mortality rates, according to the literature [35]. Thus, the Brazilian National Health System must guarantee comprehensive and horizontal care in emergency situations [36], with emphasis on comprehensive care, monitoring of vulnerable families and follow-up for suspected and mild cases, these being fundamental strategies both for containment of the pandemic, and also for the condition of people with covid-19 not becoming worse [37]. However, with the dismantling of the social protection system, devaluation and disinvestment in science, technology and education, and the precariousness of public health services, greater difficulties arise for guaranteeing quality of care at all levels [37, 38]. Several studies point out that groups with higher rates of social vulnerability are subject to worse covid-19 outcomes, which include worse complications arising from the disease, longer hospital stays and even higher occurrence of deaths, and these aspects may be associated with problems with health records [39–43].

It is worth noting that Brazil is known to be a country with persistent and expressive socioeconomic inequalities, marked by income discrepancies at extreme levels, which infers that the country is one of the most unequal in the world [44]. Such inequities directly reflect on people’s quality of life, as well as access to health services, including during the pandemic period, where people with lower levels of income and schooling, who do not have health insurance and are in a vulnerable situation, are the most affected [45–47].

Indeed, inequality is a major factor when it comes to the health of the population. Several studies, as well as those already presented and discussed, focus on evaluating the impact of these inequities regarding access to and use of health services. However, the scientific literature lacks studies that assess the magnitude of these inequalities for health promotion and disease prevention indicators. Understanding counseling as a powerful health promotion strategy, both existing policies and health references recognize its effectiveness, support its need and reinforce the commitment of all professionals to providing health education actions to the population in all care spaces, with the purpose of promoting health, preventing diseases and contributing to comprehensive and equitable care [9, 10, 48]. Despite having found differences in health counseling in relation to income, no disparities were observed in other indicators, such as education. However, it is important to highlight that the discussion about health inequalities extends to a broader spectrum, which refers to the unequal distribution of resources and opportunities among different social and economic groups, which can lead to significant disparities in terms of access to healthcare services, as well as well-being and quality of life [49–51]. During the pandemic, for example, people with lower income and education levels, who do not have health insurance and are in a vulnerable situation, were the most affected, highlighting how these socioeconomic inequalities manifest themselves in complex and comprehensive ways in various dimensions of people’s lives in Brazil [45–47].

According to data released by the Oswaldo Cruz Foundation (Fiocruz) in 2020, approximately 162 million individuals rely exclusively on the Brazilian Unified Health System (SUS) [52, 53].Additionally, information from the National Health Agency (ANS) indicates that about 50.5 million people have private health insurance in the country [54]. However, access to healthcare services is more readily available for those who have private health insurance, making it more challenging for the portion of the population that relies solely on SUS [45, 46]. Therefore, access to healthcare services is influenced by individuals’ purchasing power, who have greater ability to afford private health insurance [55, 56]. As professional counseling can occur in both the public and private sectors, it is observed in the literature that those with health insurance tend to receive more guidance and professional counseling [45], which strengthens the findings of the present study.

However, the results found in this study should be interpreted considering their limitations and potential. Memory bias should be considered, as there may be underestimation of the prevalence of the outcomes. One of the strengths to note is that our sample of individuals with COVID-19 were diagnosed by the gold standard test (RT-PCR), with a high response rate (> 75%). In addition, this study is unprecedented in the literature and the inequality analysis allows for the identification of disparities, taking into account the entire distribution of data and not just groups at the extremes.

## Conclusion

The prevalence of counseling for preventive measures and measures to combat covid-19, physical activity, healthy eating, healthy sleep, vaccination for covid-19, healthy lifestyle, prevention of covid-19 and all the guidelines was 20.1% to 56.3%. The prevalence of all counseling increased according to income, except counseling for protective measures. The odds of receiving counseling were higher in the richest groups for healthy lifestyle, healthy eating, physical activity, healthy sleep, covid-19 prevention, vaccination and for all guidelines. There are pro-rich inequities in all health counseling with respect to income, except counseling for protective measures.

These results serve as a basis for aggregating public health promotion policies, in addition to reinforcing health counseling as an assignment of the multidisciplinary team. It is important to carry out training actions for health teams so that all individuals receive the necessary guidance/counseling, especially in primary care, with a view to promoting greater equity in health.

The study was carried out with financial support from FAPERGS - Research Support Foundation of Rio Grande do Sul, Brazil grant number 21/2551-0000107-0 Research Program for the SUS: shared management in health - PPSUS).

## Electronic supplementary material

Below is the link to the electronic supplementary material.

Supplementary Material 1

## Data Availability

Datasets used and/or analyzed during the current study are available from the corresponding author upon reasonable request.

## List of abbreviations

SUS: Unified Health System
RT-PCR: Reverse transcriptase reaction- Polymerase chain reaction
