# Supplementary material for: Inequalities in the receipt of healthcare practitioner counseling for adults after COVID-19 in southern Brazil
Source: BMC Public Health. 2023 Jun 7;23:1101. doi: 10.1186/s12889-023-15914-2 (PMC10245350; doi:10.1186/s12889-023-15914-2)
Supplement: Supplementary file 1 — Supplementary Material 1 [file 12889_2023_15914_MOESM1_ESM.docx]

| **Supplementary Table 1**. Adjusted analysis of counseling and sociodemographic variables of individuals infected by COVID-19 (December 2020 to March 2021) in the municipality of Rio Grande, Rio Grande do Sul, 2022 (n=2919). | | | | | | | | |
| --- | --- | --- | --- | --- | --- | --- | --- | --- |
|  | **Healthy eating** | **Physical activity** | **Healthy sleeping** | **Healthy lifestyle** | **Coronavirus protective measures** | **vaccination** | **COVID-19 Prevention** | **All Guidelines** |
| **Sex** |  |  |  |  |  |  |  |  |
| Female | 1 | 1 | 1 | 1 | 1 | 1 | 1 | 1 |
| Male | 0.91  (0.82; 1.01) | 0.83  (0.72; 0.95) | 0.88  (0.77; 1.00) | 0.85  (0.73; 0.99) | 0.99  (0.92; 1.07) | 0.98  (0.88; 1.09) | 1.01  (0.89; 1.14) | 0.76 (0.65;0.89) |
| **Age Group** |  |  |  |  |  |  |  |  |
| 18 to 59 years | 1 | 1 | 1 | 1 | 1 | 1 | 1 | 1 |
| 60 years or more | 0.97  (0.83; 1.14) | 1.02  (0.84; 1.22) | 1.04  (0.87; 1.23) | 0.99  (0.80; 1.24) | 0.95  (0.85; 1.06) | 1.12  (0.97; 1.29) | 1.08  (0.92; 1.26) | 0.99  (0.79; 1.26) |
| **Skin color** |  |  |  |  |  |  |  |  |
| White | 1 | 1 | 1 | 1 | 1 | 1 | 1 | 1 |
| Black/Brown | 0.98  (0.86; 1.11) | 1.02  (0.88; 1.18) | 0.95  (0.83; 1.10) | 0.96  (0.80; 1.14) | 1.04  (0.96; 1.13) | 1.01  (0.90; 1.12) | 1.04  (0.91; 1.18) | 1.07  (0.89; 1.29) |
| **Marital status** |  |  |  |  |  |  |  |  |
| Married/lives with partner | 1 | 1 | 1 | 1 | 1 | 1 | 1 | 1 |
| Single/separated/widowed | 1.00  (0.89; 1.11) | 0.97  (0.85; 1.10) | 0.93  (0.82; 1.05) | 0.87  (0.75; 1.01) | 1.04  (0.97; 1.12) | 1.01  (0.91; 1.12) | 1.00 (0.90;1.12) | 0.91  (0.77; 1.07) |
| **Education** |  |  |  |  |  |  |  |  |
| Never studied | 1 | 1 | 1 | 1 | 1 | 1 | 1 | 1 |
| Elementary education | 0.80 (0.45;1.44) | 0.75 (0.38;1.48) | 0.80 (0.41;1.57) | 0.53 (0.27;1.03) | 0.93  (0.60; 1.44) | 0.73  (0.44; 1.23) | 0.67  (0.40; 1.12) | 0.42  (0.23; 0.77) |
| High school education | 0.80 (0.44;1.44) | 0.73 (0.38;1.45) | 0.85 (0.43;1.67) | 0.61  (0.31; 1.19) | 0.85  (0.55; 1.32) | 0.73  (0.44; 1.23) | 0.65  (0.39; 1.09) | 0.43  (0.23; 0.79) |
| Higher education | 0.72 (0.40;1.30) | 0.80 (0.40;1.59) | 0.81 (0.41;1.61) | 0.59  (0.30; 1.17) | 0.88  (0.57; 1.38) | 0.76  (0.45; 1.28) | 0.64  (0.38; 1.08) | 0.43  (0.23; 0.81) |
| **Body Mass Index** |  |  |  |  |  |  |  |  |
| Low/normal weight | 1 | 1 | 1 | 1 | 1 | 1 | 1 | 1 |
| Overweight/obesity | 0.98  (0.87; 1.10) | 1.03  (0.90; 1.19) | 0.98  (0.86; 1.12) | 1.04  (0.88; 1.23) | 1.02  (0.94; 1.10) | 0.97  (0.86; 1.09) | 0.97  (0.85; 1.09) | 1.09  (0.91; 1.30) |
